# Multifactorial Mechanism of Sarcopenia and Sarcopenic Obesity. Role of Physical Exercise, Microbiota and Myokines

**DOI:** 10.3390/cells11010160

**Published:** 2022-01-04

**Authors:** Jan Bilski, Piotr Pierzchalski, Marian Szczepanik, Joanna Bonior, Jerzy A. Zoladz

**Affiliations:** 1 Department of Biomechanics and Kinesiology, Chair of Biomedical Sciences, Faculty of Health Sciences, Institute of Physiotherapy, Jagiellonian University Medical College, 31-008 Krakow, Poland; 2 Department of Medical Physiology, Chair of Biomedical Sciences, Faculty of Health Sciences, Institute of Physiotherapy, Jagiellonian University Medical College, 31-126 Krakow, Poland; piotr.pierzchalski@uj.edu.pl (P.P.); joanna.bonior@uj.edu.pl (J.B.); 3 Department of Medical Biology, Chair of Biomedical Sciences, Faculty of Health Sciences, Institute of Physiotherapy, Jagiellonian University Medical College, 31-034 Krakow, Poland; marian.szczepanik@uj.edu.pl; 4 Chair of Exercise Physiology and Muscle Bioenergetics, Faculty of Health Sciences, Jagiellonian University Medical College, 31-066 Krakow, Poland; j.zoladz@uj.edu.pl

**Keywords:** exercise, sarcopenia, sarcopenic obesity, ageing, skeletal muscle, adipose tissue, microbiota, adipokines, myokines

## Abstract

Obesity and ageing place a tremendous strain on the global healthcare system. Age-related sarcopenia is characterized by decreased muscular strength, decreased muscle quantity, quality, and decreased functional performance. Sarcopenic obesity (SO) is a condition that combines sarcopenia and obesity and has a substantial influence on the older adults’ health. Because of the complicated pathophysiology, there are disagreements and challenges in identifying and diagnosing SO. Recently, it has become clear that dysbiosis may play a role in the onset and progression of sarcopenia and SO. Skeletal muscle secretes myokines during contraction, which play an important role in controlling muscle growth, function, and metabolic balance. Myokine dysfunction can cause and aggravate obesity, sarcopenia, and SO. The only ways to prevent and slow the progression of sarcopenia, particularly sarcopenic obesity, are physical activity and correct nutritional support. While exercise cannot completely prevent sarcopenia and age-related loss in muscular function, it can certainly delay development and slow down the rate of sarcopenia. The purpose of this review was to discuss potential pathways to muscle deterioration in obese individuals. We also want to present the current understanding of the role of various factors, including microbiota and myokines, in the process of sarcopenia and SO.

## 1. Introduction

Ageing is determined by genetic background and is influenced by various environmental factors [1]. When almost every organ in the body is affected by the harmful effects of ageing, the most phenotypically visible changes affect body composition, primarily skeletal muscle, adipose, and bone tissues [2]. Muscle mass constitutes about 42% of body mass in adult humans, but it decreases to about 27% in older people [3]. The loss of body mass becomes clearly noticeable after reaching the age of 50 [4] and further accelerates with ageing. This condition is called sarcopenia (the Greek sárx, “flesh” and peníā” poverty”) [5]. Often, but not always, sarcopenia, especially after the age of 60 is accompanied by a large increase of body fat (sarcopenic obesity) and body mass. This type of sarcopenia is especially harmful to the human body since it directly decreases human exercise capacity as well as accelerates the rate of other age-related multi-organs dysfunctions [1]. Physical activity attenuates the rate of the ageing-related deterioration of muscle function but it cannot stop this process. In this review, we aim to present the current knowledge on the role of varied factors, including microbiota and myokines in the process of sarcopenia.

According to the current definition, sarcopenia is an age-related, progressive, and generalized skeletal muscle disorder characterized by low muscle strength (dynapenia), low muscle quantity and/or quality, and reduced functional performance [5,6]. This current definition of sarcopenia, unlike the previous ones, pays more attention to the reduction of muscle strength and physical impairment than just to the loss of muscle mass [5,6]. Muscle mass decreases with age, mainly at the expense of fast-twitch type II fibers [7]. The loss of muscle mass does not fully explain a parallel decline in muscle function; muscle mass and muscle strength decrease with age, but the decline in strength is two to five times faster than predicted from the decrease in muscle mass alone [7,8].

In addition to their prominent role in motor function, muscles are vital for metabolic homeostasis [9]. Sarcopenia is a crucial component of the frailty syndrome and leads to an increased likelihood of adverse side effects, such as falls, fractures, physical disability, and mortality [2,8] and a higher risk of insulin resistance, diabetes, and cardiovascular diseases [10,11,12,13,14,15]. As with muscle tissue, maximum bone mass is reached around age 30, remains constant in young adulthood, and slowly declines with age [16]. In women, after menopause, the rate of bone loss increases, leading to osteoporosis earlier than in men. However, at a later age, the rate of bone loss is similar for both sexes [17].

Unlike muscle and bone tissue, total body fat increases with ageing up to a certain age. Only later in very old age does the adipose tissue also decline [17]. However, a more critical ageing-related transformation is the pronounced redistribution of adipose tissue [5]. A continuing loss of subcutaneous adipose tissue (SAT) with age is accompanied by increased visceral obesity and an accumulation of adipocytes and lipids in different depots such as bone marrow, liver, and particularly skeletal muscle (myosteatosis) [5,18].

Appropriate nutrition is vital to inhibit the development of sarcopenia and maintain healthy ageing [19]. However, in the older adults, we often see so-called age-related anorexia. This phenomenon is caused by various factors, including loss of appetite, changes in taste, and changes in the digestive tract. It may lead to a decreased consumption of protein and calories and, consequently, a decrease in the synthesis of muscle proteins [20]. On the other hand, excessive caloric intake, leading to obesity, can also contribute to the development of sarcopenia [21].

Sarcopenic obesity (SO) occurs when a decrease in lean body mass is accompanied by an excessive accumulation of adipose tissue, especially visceral fat. The risk and incidence of SO increase with age [5]. The increasing incidence of SO and its serious consequences make it a significant health burden in an ageing population due to the frequency of serious complications [22]. Both obesity and sarcopenia are characterized by a subacute, chronic pro-inflammatory state (low-grade inflammation) that affects metabolic processes, disrupting the functioning of both adipose and skeletal muscle tissue [23]. Therefore, it is possible that sarcopenic obesity can cause much more severe health consequences than obesity or sarcopenia alone. [24]. The phenomena of sarcopenia and obesity in this way could reinforce each other in the vicious cycles of loss of muscle mass and function, growth of visceral fat, and metabolic disorders [21]. Regrettably, there is at present no clear definition of this state [5]. The coexistence of obesity with sarcopenia accelerates muscle mass and function loss, reduces physical performance, and increases mortality risk [24]. Both obesity and the ageing process contribute to the ectopic deposition of adipose tissue in skeletal muscles and other organs [18,22,25,26,27]. In addition to further muscle dysfunctions, this phenomenon contributes to other disorders: oxidative stress, inflammation, mitochondrial dysfunction, and insulin resistance [18,25]. It is worth noting that the term “osteosarcopenic obesity” has recently been proposed to emphasize the importance of excessive obesity in the deterioration of muscle and bone health [28].

Since Gruberg et al. [29] came up with the term “obesity paradox” to describe their observation that obese patients with coronary heart disease outperform their counterparts with normal body weight, many articles have described this phenomenon in various diseases. Therefore, it was wondered whether obesity could also be a protective factor in older adults, including those suffering from sarcopenia and osteosarcopenia. (for a review, see Bosello and Vanzo, 2021) [30] 

A recent study by Bahat et al. [31] confirmed that in older adults with sarcopenia, obesity might have a protective effect against the limitations of some functional measures. Perna et al. [32] proposed the existence of two phenotypes: osteosarcopenic visceral obesity (OVO) and osteosarcopenic subcutaneous obesity (OSO). They have shown that the visceral obesity form is much more common and that older patients suffering from OVO have a greater risk of fractures, inflammation and metabolic disorders than those with OSO. Moreover, patients with OSO seem to benefit from this type of obesity, in line with the often described “obesity paradox” [30,32]. It was also suggested that while subcutaneous adipose tissue may be responsible for these beneficial effects, ectopic obesity, especially peri-muscular fat, may have adverse health consequences. Interestingly, it was also demonstrated that adipokines and myokines would play a significant role in both beneficial and adverse effects of adipose tissue [33,34,35].

## 2. Pathomechanism of Changes in Skeletal Muscle in Sarcopenia and Sarcopenic Obesity

**Figure 1:**
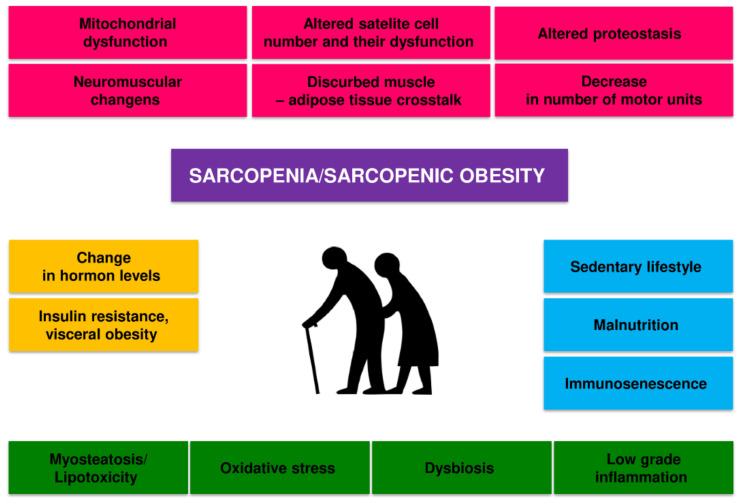
Potential pathogenic mechanisms of age-related sarcopenia and sarcopenic obesity.

Sarcopenia is caused by a combination of factors, including neurological factors associated with loss of motor neurons, loss of muscle motor units, endocrine changes, and lifestyle changes associated with sedentary behavior and poor nutrition [36,37,38,39] (Figure 1).

There is a dynamic balance in the body between the synthesis and degradation of muscle proteins. Muscle hypertrophy occurs when the synthesis of proteins exceeds their breakdown, and skeletal muscle atrophy occurs when the breakdown is dominant. The mechanisms of development of sarcopenia and SO are diverse, complex, and not fully understood. Several factors can influence the development of sarcopenia in the older adults, including hormone and cytokine imbalance, age-associated systemic inflammation (inflammaging), gut microbiota dysbiosis, microcirculation disorders, metabolic disorders, predominantly obesity and insulin resistance [36,37]. Old age-related physical inactivity and quantitative and qualitative malnutrition will also contribute to this process [38,39].

All these factors can interact in a complex way on skeletal muscle, reducing the expression of skeletal muscle growth factors and increasing oxidative stress, and the activity of the ubiquitin-proteasome system and autophagy [36]. These mechanisms disrupt the balance between the synthesis and breakdown of muscle proteins, lead to a decrease in the number and function of satellite cells and dysfunction of mitochondria, and ultimately to atrophy and dysfunction of skeletal muscles [36,37,40]. The disorders of the nervous motor system and its interaction with skeletal muscles also play a significant role [41]. The loss of alpha motor neurons and disorders of neuromuscular connections contribute to the disappearance of muscle fibers, especially Type II fibers, and the transition of Type II muscle fibers to Type I muscle fibers [36,37]. Changes in the structure and function of the neuromuscular junction with ageing also contribute considerably to sarcopenia [42]. These issues have recently been extensively discussed [37,42]. In general, it is considered that the main factors that contribute to the loss of muscle power generating capabilities during ageing are as follows: (i) loss of muscle mass; (ii) fast-to-slow transition in areal fiber type composition; (iii) an increase in connective tissue; and (iv) altered neural drive (for review see, Degens, 2019) [4].

**Figure 2:**
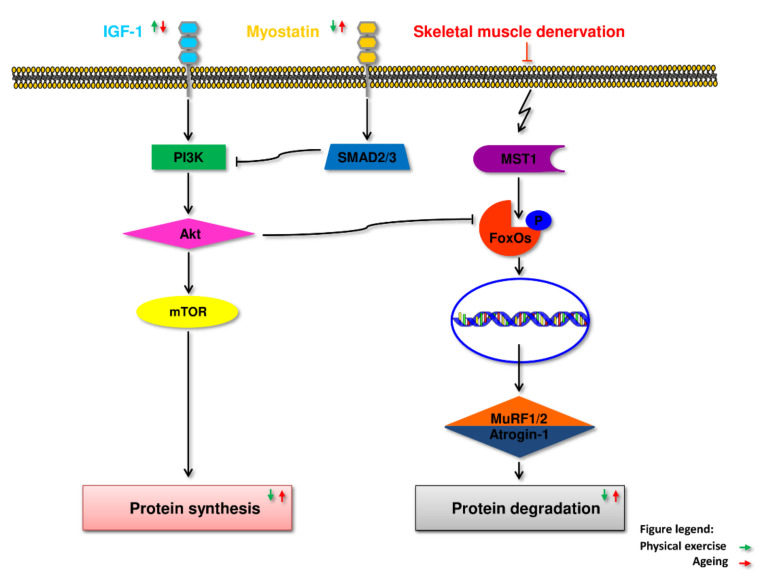
Diagram illustrating myostatin, and IGF-1 pathway interactions. Myostatin’s effects require both Smad2 and Smad3, which block muscle differentiation. Smad2 and 3 activations are both required for myostatin’s inhibitory effects on Akt. IGF-1 released in response to exercise can counteract myostatin’s effects.

Maintaining skeletal muscle mass and function is multifaceted and depends on complex regulatory processes in response to ageing, disease and injury, exercise and diet [43]. These processes include the process of myogenesis and, in particular, the activation of satellite muscle cells and proliferation of myoblasts; the withdrawal of myoblasts from the cell cycle, their subsequent differentiation and fusion into multinucleated muscle fibers [44,45]. They also include the processes of repair and reconstruction of muscle tissue [46,47] and balance between the breakdown of skeletal muscle proteins and their synthesis [48]. A signaling system including growth factors such as insulin-like growth factor 1 (IGF1) and a cascade of intracellular components plays a vital role in regulating skeletal muscle growth. The Akt kinase, also known as protein kinase B (PKB), is the central component of this cascade, controlling both protein synthesis via the mammalian target of rapamycin (mTOR), also referred to as rapamycin mechanistic target, and glycogen synthase kinase 3 (GSK3), and protein degradation via transcription factors of the FoxO family [49]. Activation of this pathway is essential to induce load-induced skeletal muscle hypertrophy. mTOR is present in at least two multi-protein complexes known as mTORC1 and mTORC2. mTORC1, a raptor-binding protein, can stimulate protein synthesis (Figure 2). Increased protein synthesis and hypertrophy necessitate increased ribosome activity, which can be done via increasing ribosome efficiency (i.e., more mRNA translation to the ribosome) and/or ribosome capacity (through ribosome biogenesis) mTORC1 activity regulates both processes at least partially [49]. The sarcopenic muscles have impaired this pathway, which may play a role in the development of sarcopenia [50].

Muscle stem cells, also known as satellite cells, play a crucial role in muscle fiber regeneration, repair, and muscle hypertrophy. Satellite cells are found under the basal lamina of muscle fibers and are mitotically quiescent in adult life [51]. When a muscle is injured, the dormant satellite cells are activated, which leads to their proliferation and differentiation into myoblasts [51]. With age, the regenerative abilities of ageing muscles gradually deteriorate. The number of satellite muscle cells, especially satellite type II, clearly decreases, and their function is impaired, leading to the accumulation of unrepaired muscle cells [45,51].

The way in which the size of individual organs of our body is so precisely controlled remains a complex biological problem. During an organism development, all its elements are subject to many changes, including changes in size. It has been known for some time that the control of the size of individual organ takes place to a certain extent in an autonomous way, but at the same time while maintaining a proper proportion to other body organs. This phenomenon has been confirmed in classic experiments carried out on the mouse spleen and thymus model by Metcalf [52,53]. This applies to all organs, in particular muscles. The dynamics of changes in the size of human skeletal muscle are associated with many variables as age, gender, sex hormones level, intensity of training or nutrition as it has been described above.

**Figure 3:**
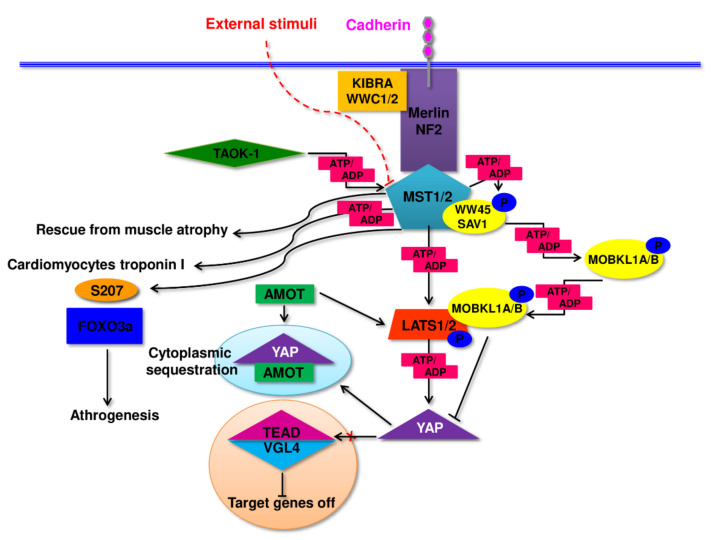
A general outline of the functioning of the Hippo pathway. Diagram presents central axis of pathway consisting of ‘core kinases’ MST1/2, LATS1/2, their up-stream modulators: Merlin/NF2, Kibra/WWC1/2 and effector part of the pathway—YAP (Yes-associated protein encoded by YAP1). What is worth mentioning is active ‘core kinases’ phosphorylate YAP resulting in its cytoplasmic sequestration and down-regulation of the pathway activity. A detailed description of the pathway functioning is in the text of the article. MST1/2 (mammalian sterile 20-like kinases), LATS (large tumor suppressor kinases), Vgll1-4 (vestigial-like, Vito, Tondu), Tead1-4 (TEA/ATTS domain/TEF/scalloped), Merlin/NF2 (neurofibromatosis type 2), WW45/Sav1 (adaptor proteins Salvador homologue1), MOBKL1A (Mps-one binder kinase activator 1), TAOK1, thousand-and-one amino acids kinase 1, Amot (angiomotin). FOXO3a (Forkhead box O3).

Hippo pathway is responsible for the control of the growth processes, partly cell cycle and apoptosis in all eukaryotic cells. This unique pathway integrates signals from many surface receptors and other internal molecular signal to manage regeneration and cell division processes. In a brief, Hippo pathway constitute of central ‘core kinases’ which interact with specific adhesion molecules via up-stream modulators (Figure 3). The effector part contains a target of the core kinases: transcriptional activator Yes-associated protein (YAP) and transcriptional co-activator with PDZ-binding motif (TAZ). The functioning of this signaling pathway shows a specific reversal mode of its main elements. If the core Hippo kinases remain active and their effector proteins are phosphorylated, there is no interference with genes being under Hippo control. Paradoxically, nuclear translocation of active YAP/TAZ complex occurs in an un-phosphorylated form and activates or suppresses transcription factors involved in the regulation of genes controlling cell proliferation, tissue growth, and organ size [54,55]. Due to a lack of intrinsic DNA-binding domains in YAP/TAZ complex its members have no ability to direct binding and influencing the promoters of regulated genes. Thus, the only way to influence the expression of controlled genes is an interaction with others directly binding with DNA nuclear factors. Due to this mechanism YAP/TAZ can target a variety of different transcription factors and in this circuitous way control genes responsible for growth and cell viability. Active—unphosphorylated YAP as well as TAZ complexed with Vgll1-4 (vestigial-like, Vito, Tondu) act as a co-activator of Tead1-4 (TEA/ATTS domain/TEF/scalloped) using specific Tead-co-factor binding domains [56,57].

Activation of “core kinases”: MST1/2 (mammalian sterile 20-like kinases), LATS (large tumor suppressor kinases), occurring due to co-localization to the cellular membrane, results in phosphorylation of YAP-1 and its consecutive cytoplasmic sequestration or degradation, in consequence, down-regulating of the entire Hippo pathway activity [58,59]. The process of membrane “anchoring” of the Hippo pathway core kinases utilizes multiple intermediary proteins like Merlin/NF2 (neurofibromatosis type 2) employing Kibra/WWC1/2 proteins. Assembling this membrane docking complex might occur in reaction to other signaling pathways members as STATS (Signal Transducers and Activators of Transcription), PI3K (phosphatidylinositol 3-kinase), RAS or receptors, adhesins such as CD44, cadherins or other receptors participating in cellular connections. In some cases, the complex might be extended to the Kibra-FRMD6/Ex1-Merlin/NF2 form. To sustain and enhance the activity of MST1/2 kinases, WW45/Sav1 (SAV1/WW45, adaptor proteins Salvador homologue1) might be employed as a connector of “core-kinases” complexes or TAOK1, thousand-and-one amino acids kinase 1 as a direct activator of MST1/2, through the phosphorylation. In this reaction TAOK1 acts as MAP3K (mitogen-activated protein kinase kinase kinase) [60,61,62,63]. Activated MST1/2 kinases tend to phosphorylate their substrate protein MOBKL1A (Mps-one binder kinase activator 1). Phosphorylated MOBKL1A acquires the ability to bind to LATS1/2 kinases, physically covering their auto-inhibitory motif. This action becomes the inducing signal for LATS kinases increasing their activity and eventually phosphorylating YAP. The most effective way of the abolishment of YAP-1 activity— LATS dependent phosphorylation of HxRxxS motif at Ser 381 what triggers follow-up phosphorylation by casein kinase 1 (CK1 delta/epsilon) at Ser 400 and Ser 403 leading to consecutive ubiquitin-mediated degradation. Analogically, TAZ is phosphorylated at S311 by LATS1/2 and at Ser 314 by casein kinase 1 with the same effect as YAP— degradation. As it has been mentioned, phosphorylated YAP become inactivated, and this might also be achieved in other ways: i). by binding with 14-3-3 protein; ii). by its cytoplasmic sequestration in complex with angiomotin (Amot) and angiomotin-related AmotL1 and AmotL2—its, as well as TAZ, negative regulators [64,65,66,67].

Hippo signaling down-regulates the proliferation stimuli and loss of MST1/2 activity or overexpression of YAP-1 might result in tissue overgrowth. Moreover, loss of MST1 or MST2 gene function due to mutation, leads to an instant raise of mTORC1 (mTOR complexes 1) but not mTORC2 activity, confirming the involvement of both pathways, Hippo and mTOR (mammalian target of rapamycin serine/threonine protein kinase), in the monitoring of cell growth and organ size [68].

The member of PI3K superfamily of kinases, mTOR, is a precise sensor of signals related to cellular metabolism (amino acid and glucose level), but also detects signals associated with cell stress (heat shock, hypoxia, DNA damage or oxidative damage related to exposure to oxygen radicals). Such a precise response is possible due to the structural diversity of two basic mTORC1 and mTORC2 complexes, which achieved a significant specialization in detecting and transferring signals regulating the vital to the cell processes [69]. mTORC1 is composed of PRAS40 (proline-rich AKT substrate 40 kDa); mLST8 (mammalian lethal with Sec13 protein 8); Raptor (regulatory-associated protein of mTOR) and Deptor (DEP-domain-containing mTOR-interacting protein) and is mainly involved in controlling such processes as protein synthesis through supervision over phosphorylation EIF4E-Binding Protein 1 and the P70 Ribosomal S6 Kinase 1 or lipid synthesis through governing the activity of SREBP1 (sterol regulatory element binding protein 1) and PPARγ (peroxisome proliferator-activated receptor-γ). To some extend mTORC1 pathway oversee an inflammatory response or reaction to WNT (wingless-related integration site) signaling [70,71,72]. The function of mTORC2 seems to be more focused on the supervision of proliferation control. mTORC2 is one of two kinases which in simultaneous manner phosphorylate AKT-1 to let it reach full activation. mTORC2 is responsible for selective phosphorylation of Akt Ser 473 whereas PDK-1 (phosphoinositide-dependent kinase 1) for phosphorylation of Ser 308 [73]. Muscle hypertrophy in response to endurance exercise training seems to be regulated via mTORC1 through its ability of stimulation of protein synthesis followed by refiber phenotype shift and acceleration of mitochondrial biogenesis [74].

**Figure 4:**
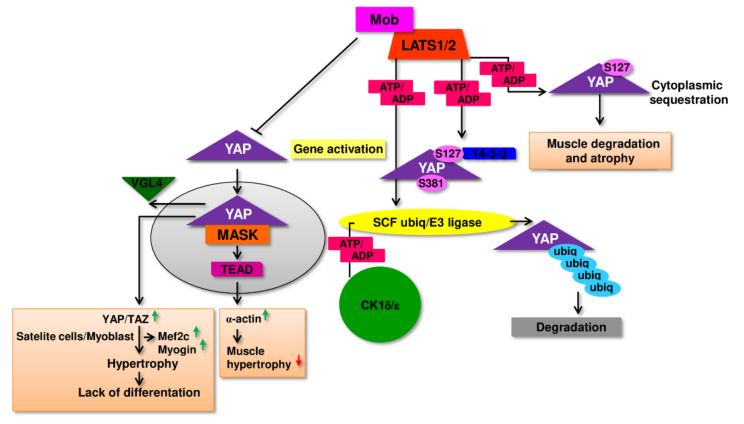
Involvement of Hippo signaling in muscle cells development. Active— non phosphorylated YAP nuclear translocation results in direct activation of co-activation of TEAD dependent genes regulating muscle cells such as α-actin, Mef2c and Myogin. For details, please refer to the text.

The growing volume of experimental evidence indicates that for the regulation of physiological and pathological changes taking place in muscle tissue might also be responsible the Hippo pathway [75,76]. Carson et. al. [77] confirmed that direct target protein of YAP in the regulatory complexes, Tead1, up-regulate a-actin gene expression in chicken muscle during hypertrophy. This observation may indicate, that in muscle cells, Hippo and mTOR cooperate, where Hippo controls myofibrillar gene transcription during muscle hypertrophy and mTOR is accountable for increased translation and protein synthesis (Figure 4). This type of cooperation is often described in the comprehensive regulation of cellular processes. As a support of this thesis might be the observation that in fetal fast-twitch mouse muscle the expression of YAP protein is very high with tendency to declining in post-natal period [78]. On the other hand, Judson et. al. [79] has demonstrated that overexpression of constitutively activated through specific phosphorylation S127A YAP-1 in mouse skeletal muscle resulted in its degradation, and atrophy. No myofiber hypertrophy or any muscle fiber shift from fast to slow has been observed. Thus, however the observed phenomenon was reversible, we can hypothesize that high activity of Hippo pathway might be rather connected to the process of muscle atrophy and eventual sarcopenia [79]. Similarly, total YAP level was found to be upregulated during atrophy of muscle because of denervation in SOD-1G93A mouse model of amyotrophic lateral sclerosis as well as in mdx mouse model of Duchenne muscular dystrophy [80].

**Figure 5:**
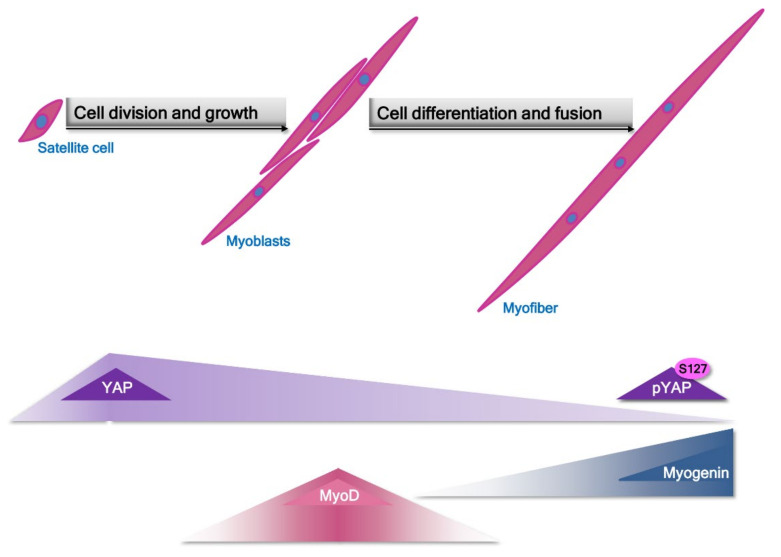
Diagram presenting dynamics of changes in expression of YAP, MyoD and myogenin during activation of satellite cells division, conversion to myoblast and differentiation to myofiber. Based on the information summarized in the article, Hippo pathway might be considered an additional important mediator of balance between development and differentiation of muscle cells.

Additionally, some researchers reported that expression of YAP protein reached 2-fold higher level in slow-twitch muscle refibers than in fast-twitch isolated from young subjects. Moreover, total YAP expression tends to decrease by 50% in the aged subject when compared to young ones. These data might imply a role of YAP in the age-dependent sarcopenia [81]. Some other information reveals that the expression and activity profile of the Hippo signaling pathway elements can also depend on the type of muscle cells. For example, in satellite cells/myoblasts YAP and TAZ activity are increased, and its excessive activity due to over-expression of mutant YAP/TAZ unable to be inhibited by Lats1/2 lead to heightened proliferation of these cells and at the same time this elevated level of YAP activity makes impossible to launch terminal differentiation program which is essential for fusion and proper myofibers formation (Figure 5). In these cells substantial decrease of two genes involved in differentiation -myogenin and Mef2c but Myf5, myoblast proliferation related gene retained a high level of expression.

Moreover, employing microarray analysis authors established that many of the cell cycle regulators and myogenic differentiation factors were targeted by YAP via specific binding with TEAD leading to activation of muscle-specific cytidine-adenosinethymidine (MCAT)-elements in myoblasts [82,83]. From the other hand, inhibition of Mst1/2 kinases activity seems to be sufficient to rescue from atrophy denervated fast-twitch muscle, suggesting that stimuli initiating the canonical Hippo response might result in anabolic/catabolic response in skeletal muscle [84]. To complicate the above-mentioned image of the Hippo pathway participation in the development of muscle cells, Sun et al. [85] presented evidence that in mice model as well as in vitro experiments TAZ and YAP shared activity promoting proliferation but along the myogenesis process the activity of both factors became opposite. When TAZ tend to enhance differentiation of myoblasts YAP inhibited this process, showing clearly that activity of YAP and TAZ overlap during proliferation but contradict during myogenic differentiation [85].

Another component of the Hippo pathway, “core-kinases” MST1/2 seem to also participate in the regulation of muscle specific gene expression and direct phosphorylate some of muscle specific proteins. In cardiomyocytes was found to co-localize with troponin I and phosphorylate it in vivo [86]. In hypertension caused hypertrophy or myocardial infarction MST1 was found to be activated in cardiomyocytes inducing apoptosis of these cells [87]. Similarly, in skeletal muscle cells MST1 reacted with rapid activation upon its denervation. This activation of MST1 resulted with Ser207 specific phosphorylation and consecutive nuclear translocation of FOXO3a transcription factor, general activator of atrogenes. The process was followed with muscle atrophy [84].

From the information provided above we can conclude that Hippo signaling is involved in the regulation of physiological phenomena and pathological reactions in muscle cells. Many conflicting observations regarding its participation in myogenesis and sarcopenia do not allow for unambiguous determination of its role in these processes.

Recently, much importance has been attached to intramuscular fat infiltration, which leads to further disorders of the structure and function of skeletal muscles and the development of sarcopenia [18,36].

Although the mechanisms underlying sarcopenia and its consequences are still not fully understood, chronic inflammation and immune disorders are essential. Although still not fully defined, the concept of immunosenescence is used to describe the totality of age-related changes leading to the deterioration of the functional state of the immune system [1,88,89,90].

In the process of ageing, the immune cell secretion profile is altered, increasing the release of pro-inflammatory cytokines and developing “inflammaging, occurring in the absence of infection (sterile inflammation), leading to tissue damage [23,89]. The pro-inflammatory cytokines may contribute to the development of sarcopenia by activating the ubiquitin-protease system [91,92]. They may also antagonize the pro anabolic effects of insulin growth factor-1 (IGF-1) [93,94]. Inflammaging may also be responsible for anabolic resistance, and the fact that skeletal muscle protein biosynthesis in response to physiological stimuli is insufficient to maintain skeletal muscle in older adults [95].

Chronic low-grade inflammation associated with obesity and the ageing process may affect the simultaneous development of insulin resistance (IR) and anabolic resistance (AR) [23,96,97,98]. The latter is understood as an impaired synthesis of skeletal muscle proteins to anabolic stimuli such as dietary proteins or physical activity [99,100,101]. Therefore, together, IR and AR can act synergistically, lead to disturbances in adipose tissue metabolism, skeletal muscles and bones, and contribute to the development of type 2 diabetes (T2D) and osteosarcopenic obesity [98,102]. Both low-grade generalized inflammation and intramuscular fat infiltration can lead to mitochondrial dysfunction and impaired myokine release [103].

As the interaction between the immune cells and skeletal muscles is essential for the proper regeneration of the latter, it is clear that immunosenescence can influence skeletal muscle repair [90]. In injury, immune cells infiltrate skeletal muscle and function by removing necrotic cells and secreting growth factors influencing satellite cell proliferation and differentiation [104]. During ageing, the process of immunosenescence leads to the loss of normal function of these cells and impaired regeneration of skeletal muscles [105].

Proper regeneration of skeletal muscle requires local expansion of a particular population of CD4+ CD25+ Foxp3+ regulatory T (Treg) cells [106], which is depleted in older mice [107]. Since abnormal macrophage polarity was reported in ageing mice [108], it was hypothesized that the impaired activity of M2 macrophages might be at least partially responsible for the inflammatory response and skeletal muscle atrophy during ageing [109]. The interplay of the immune system and the skeletal muscles is not one-sided [109,110]. Skeletal muscles play a crucial role in maintaining body posture and locomotion but are also an organ that can influence the body’s overall function, regulate metabolism, and modulate immune function [109,110,111].

Interestingly, declining immune function in older adults is also associated with dysbiosis. A direct link between age-related dysbiosis and age-associated systemic inflammation has been shown in a study in which cohousing germ-free (GF) mice with old, but not young, conventionally raised mice increased intestinal permeability and pro-inflammatory cytokines in the blood leading to age-related inflammation [112].

Although microbes reside in several anatomical locations, colonizing all surfaces covered by epithelia such as the skin, vagina, airways, and mouth, the lower gastrointestinal tract of mammals harbors the greatest density and diversity of commensal microorganisms. These include bacteria, archaea, fungi, viruses and protozoans. Bacteria, however, predominate and reach 1014 microbial cells in the colon [113].

Interestingly, microbial communities have been even isolated from formerly “forbidden” niches, formerly considered sterile, such as the placenta, breast, uterus, Fallopian tubes and even semen [114]. Similar to mucosal surfaces, the skin also is populated by microbiota. Like other mammals, a human infant emerges into the world from an almost sterile environment, and that in the first months of life, the infant is colonized gradually by bacteria. There are two periods of intestinal colonization: the phase of milk nourishment consumption and the stage of changing from a milk diet to solid foods [115]. The rapid development of bacterial diversity observed in the first year of life slows significantly by 3 years of age and at the age of 7–12 years starts to resemble the microbiota in adults [116], but microbial communities at this age taxonomically and functionally distinct from those of adults [117]. In adults, the human gut microbiota is composed predominantly of *Bacteroidetes* and *Firmicutes* (90%), complemented with Actinobacteria, *Proteobacteria* and *Verrucomicrobia* [118] and is relatively consistent across healthy individuals. Studies conducted over many years have shown that the bacteria living in the alimentary tract have an essential role in the processes of food digestion, production of vitamins, a transformation of xenobiotics, promotion of angiogenesis, immunity to infections, and maintenance of immune homeostasis [113]. In addition, the gut microbiota is involved in host metabolism by contributing to bile acid metabolism and recirculation, absorption of iron, magnesium and calcium, and regulation of fat storage [119]. Moreover, it has been shown that gut microbiota is a source of various bacterial products and metabolites that breach the intestinal epithelium. Short-chain fatty acids (SCFAs) such as acetate, propionate, and butyrate are widely recognized modulators of immune response in the periphery, produced during bacterial fermentation of indigestible polysaccharides [113]).

Interestingly, in the older adults, gut microbiota becomes unstable and less diverse what has been linked with increased frailty and deterioration of the immune system [120]). It is believed that observed in older adults, low gut microbiota richness is a predictor of morbidity and mortality, whereas enrichment of certain bacteria, for example, *Akkermansia* and *Bifidobacterium* is associated with longevity [120]. Among the age-associated changes in microbiota, a reduced abundance of several butyrate producers (*Clostridium clusters XIVa* and *IV*) has been found [121]. This is accompanied by the expansion of Proteobacteria and other opportunistic microbes such as *Ruminococcaceae, Fusobacterium* and *Parabacteroides*, which are present in low abundance in healthy adults [122].

Age-associated dysbiosis, thinning of the mucin layer, and increased epithelial gaps are responsible for increased mucosal barrier permeability, which allows the translocation of microbes and microbial products into the circulation [122]. Animal studies suggest that translocation of microbes and microbial products, termed pathogen-associated molecular patterns (PAMPs), from the gut lumen into the circulation is an important factor contributing to age-associated systemic inflammation and immune system dysregulation involved in numerous age-related diseases in humans [23,112]. Moreover, it is worth noting that age-associated dysbiosis could promote not only inflammaging but anabolic resistance as well, ultimately conditioning reduced muscle size, impaired muscle function and adverse clinical outcomes [123]. Numerous animal studies show that intestinal microbiota can regulate skeletal muscle function. It is worth noting that GF mice devoid of all microorganisms have lower muscle mass and fewer muscle fibers, whereas muscle atrophy markers are elevated compared to specific pathogen-free (SPF) mice. Observed changes were reversed after fecal microbiota transplantation (FMT) and SCFA supplementation [124].

Further, FMT from older adults (high-functioning group) and (low-functioning group) people into GF mice showed that the grip strength was significantly increased in high-functioning when compared with low-functioning mice [125]. The data of this animal study are supported by a randomized controlled, double-blind study showing that prebiotic supplementation increases the grip strength in older people [126]. The other animal studies show that supplementation with *Faecalibacterium prausnitzi* increases muscle mass compared to the control group [127].

Over recent years, probiotics have been used in athletes because of their improved performance and reduced fatigue after exercise [128]. Animal studies employing mice showed that 4-week supplementation of *Lactobacillus salivarius* could significantly improve muscle strength and endurance, increase liver and muscle glycogen storage, and acid kinase after exercise [129]. Interestingly, FMT containing *Bifidobacterium longum* isolated from weightlifting gold medal champion into the mice increased muscle strength and endurance together with liver and muscle glycogen storage [130]. Furthermore, it has been shown that *Lactobacillus* supplementation could improve exercise performance and significantly increase muscle mass in healthy people and significantly reduce muscle loss in cancer patients [131]. There are reports suggesting that the use of prebiotics (inulin and trans-galactooligosaccharides) has beneficial effects on skeletal muscle in mice [132].

Moreover, prebiotic (inulin and fructo-oligosaccharides) supplementation increased muscle strength and endurance in older people suggesting the beneficial influence of prebiotics on muscle function. [126]. It is suggested that supplementation with prebiotics increases the abundance of *Bifidobacterium* and butyrate producers thereby improving muscle mass and function in older people [133]. The potential involvement of gut microbiota and prebiotics in muscle function is supported by experiments showing that antibiotic treatment significantly reduces exercise endurance in mice due to the reduced ability to use glycogen for energy production [134]. Interestingly, it was observed that sarcopenia is alleviated by oral supplementation with specific *Lactobacillus* species in a mouse model of acute leukemia [135], whereas the muscle mass and function increased [136]. A limited number of animal and human studies suggest the existence of the gut-muscle axis actively involved in the pathophysiology of physical frailty and sarcopenia [137]. Factors that contribute to age-associated dysbiosis include altered diet, reduced physical activity, pharmaceuticals, altered gut morphology and reduced intestinal functionality [122]. It is still unclear how dysbiosis may contribute to sarcopenia development. It is suggested that dysbiosis affects protein metabolism, including absorption and availability reduction and increased hydrolysis, leading to reduction of muscle protein synthesis and the development of sarcopenia [137]. Moreover, gut microbiota dysbiosis contributes to gut barrier dysfunction facilitating translocation of microbial byproducts, for example, lipopolysaccharide (LPS), into the circulation, causing systemic low-grade inflammation and insulin resistance and finally leading to sarcopenia [137]. It is also possible that barrier leakiness and microbial dysbiosis in older people could activate immune cells in mucosal tissues, which migrate to the affected organs, for example, muscles in the periphery [138]. Additionally, gut microbiota dysbiosis results in reduced production of immunoregulatory and anti-inflammatory SCFAs, which could support sarcopenia development. Furthermore, SCFAs affect skeletal muscle cell function by promoting mitochondrial activity [139]. It is postulated that decreased production of SCFAs by age-modified microbiota could promote insulin resistance, decrease mitochondrial fatty acid oxidation, and support intramuscular fatty acid deposition. This leads to decreased muscle strength, insulin resistance, and sarcopenia [123].

Discussing the role of microbiota products in sarcopenia, we should not forget about toxins having negative effects, such as indoxyl sulfate. It has been observed that circulating levels of microbiota-derived indoxyl sulfate are positively associated with the expression of atroginin-1 and myostatin, which are the main negative regulators of skeletal muscle mass [123]. On the other hand, phenolic compounds produced by gut microbiota can increase glucose uptake in muscle cells, promoting anabolic responses that increase muscle mass [140]. It is worth noting that there is evidence that there is a connection between microbiota and mitochondrial function. Indeed, a decrease of butyrate production by dysbiotic gut microbiota impairs mitochondrial function [141].

Furthermore, SCFAs are the putative mediators of the effect of gut microbiota on skeletal muscle by acting on muscle mitochondria [137]. Thus, dysbiosis and reduced production of SCFAs in the older adults may contribute to the development of sarcopenia. Other studies suggest that mitochondrial dysfunction in muscle cells occurs in sarcopenia [121]. A trigger factor of inflammation in sarcopenia could be oxidized cell-free mtDNA from aged mitochondria generated in dysbiotic older people. Oxidized cell-free mtDNA as a damage-associated molecular pattern (DAMP) could activate innate immunity and promote the subsequent synthesis of pro-inflammatory mediators, which fuels sterile inflammation contributing to muscle wasting [121]. Finally, gut microbiota dysbiosis can promote “anorexia of ageing”. Indeed, microbial metabolites can act as endocrine regulators of appetite, as shown in animal models of inflammation induced by *Escherichia coli* [142]. This may suggest that, in older people, the dysbiotic gut microbiota could influence the onset of sarcopenia and physical frailty also by promotion of malnutrition [123]. Further work is required to fully understand the role of gut microbiota dysbiosis in sarcopenia development.

At least in part, mitochondrial dysfunction is linked to the ageing and obesity processes [143,144]. After the deposition of intracellular lipids, mitochondrial dysfunction and increased generation of reactive oxygen species occur in the muscles, disrupting muscle protein synthesis and impairing skeletal muscle function [145,146].

Unfortunately, due to unclear definitions of sarcopenia and the lack of accurate screening tools, most cases of sarcopenia remain unrecognized, and no intervention is undertaken. Moreover, there is no reliable and safe intervention against sarcopenia apart from increased physical activity and proper nutrition recommendations [5,38,39,147]. There is, therefore, a need to find effective biomarkers and appropriate therapeutic interventions.

## 3. Obesity

Obesity is usually defined as an excessive or abnormal accumulation of body fat that adversely affects health [148]. The adipose tissue dysfunction in the present in obesity leads to low-grade chronic inflammation, characterized by the activation of pro-inflammatory pathways and a shift in adipokine release towards a pro-inflammatory profile [149,150]. It is associated with developing metabolic and cardiovascular diseases and some cancers [151,152]. The prevalence of obesity increases with age, and in an ageing population, this obesity epidemic is a growing health care problem [148,153,154].

In mammals, adipose tissue is not homogeneous; there are two main types: white adipose tissue (WAT), which stores excess energy as triglycerides, and brown adipose tissue (BAT), which dissipates stored energy as heat [155,156].

Studies in recent years have revealed other types of adipose tissue. Of particular interest are newly identified adipocytes displaying features of both brown and white fat cells, usually developing in subcutaneous WAT from a separate subset of preadipocytes [157]. Due to their appearance and location, these adipocytes have been called brite or beige adipocytes [158]. Beige adipocytes are activated in response to cold, β3-adrenergic stimuli and peroxisome proliferators-activated receptors (PPAR -γ) in a process called adipose tissue browning. Brown and beige adipocytes seem to have different developmental origins: typical brown adipocytes come from MYF5+ (muscle developmental gene) mesenchymal stem cells in the embryonic mesoderm. In contrast, beige cells appear to come from endothelial and perivascular cells in WAT stores [159].

There are two main anatomical compartments in WAT: subcutaneous (SAT) and visceral adipose tissue (VAT), which demonstrate different metabolic and immunological profiles [160,161]. Both VAT and SAT store energy in the form of triacylglycerols and are endocrine organs that regulate energy homeostasis and metabolism. VAT also provides a protective lining for vital, visceral organs, while the subcutaneous WAT insulation against temperature fluctuations [156].

The main BAT depot is situated in the deep interscapular region extending to the subscapular, cervical, and axillary areas. BAT also is present at aortic, paraspinal, and adrenal sites. The primary function of BAT is to mediate adaptive thermogenesis in multicellular, mitochondria-rich, and UCP1)-positive brown adipocytes. Active BAT is inversely related to obesity and insulin resistance [162].

As opposed to SAT accumulation, high VAT is associated with increased metabolic and cardiovascular diseases and premature death risk [151,163]. Adipose tissue is mainly composed of adipocytes, although other cell types contribute to its growth and function, including pre-adipocytes, macrophages, lymphocytes, fibroblasts and vascular cells [164] In lean individuals, normal VAT is characterized by good vascularization, the presence of regulatory and immunosuppressive cells, such as alternatively activated (M2) adipose tissue macrophages (ATM), Treg cells, Th2 cells, eosinophils and the secretion of anti-inflammatory substances [165]. The expansion of VAT in obesity leads to the pro-inflammatory transformation associated with the secretion by adipocytes of many pro-inflammatory molecules. Adipocytes become hypertrophic, hypoxic, and die, triggering an innate immune response [165]. The hypertrophic adipocytes show reduced production of anti-inflammatory adipokines such as adiponectin. The infiltration of the adipose tissue by the pro-inflammatory immune cells such as classically activated (M1) macrophages, CD8+ and Th1 T cells and the reduced number of Treg cells further increase the production of inflammatory mediators [166]. Yap levels in the skeletal muscles of obese and insulin-resistant humans and animals were significantly lowered [167].

It is now recognized that the development of obesity is associated with alterations in the gut microbial composition. Microorganisms colonize all surfaces covered by epithelia and they occur in the greatest number in the alimentary tract. The highest species diversity is observed in the large intestine and the major groups of bacteria occurring in the intestinal lumen include *Firmicutes* (e.g., *Lactobacillus*) and Bacteroidetes (e.g., *Bacteroides*) [168]. Dysbiosis has been implicated in numerous inflammatory and autoimmune diseases, including inflammatory bowel disease, coeliac disease, rheumatoid arthritis, type 1 diabetes, multiple sclerosis, allergy and obesity. It has been shown that GF mice are resistant to diet-induced obesity, and transplantation of the microbiota from obese mice can transfer the metabolic phenotype to germ-free mice [115]. The microbiota has been proposed to affect the metabolism by promoting energy harvest through fermentation of dietary carbohydrates, regulating lipid metabolism and storage in the liver and adipose tissue, modulating secretion of enteroendocrine hormones, affecting bile acid metabolism and inducing endotoxemia and inflammation [115]. It has been reported that HFD affects the composition of the gut microbiota in mice. Direct examinations of the gut microbiota in humans and mice revealed differences associated with obesity at the phylum level, with higher numbers of Firmicutes than Bacteroidetes and less diversity overall in obese individuals compared with normal weight individuals.

Additionally, increased intestinal permeability was found in obese patients and mice kept on HFD, and it was due to a number of features, including the direct effects of the western diet and loss of bacteria, such as *Bifidobacterium* that maintain intestinal barrier function [169]. Moreover, specific Enterobacter species isolated from the colonic flora of obese individuals can induce inflammation and increased gut permeability in GF mice fed HFD. It is believed that a combination of dysbiosis and increased gut permeability is responsible for increased plasma levels of lipopolysaccharide (LPS). In addition to increased levels of LPS, low-level bacteriemia was found in most obese patients, with increased levels in those individuals who developed T2D [169]. Animal studies showed that the presence of low levels of endotoxemia has a direct role in the development of obesity. Interestingly, it seems that bacterial species associated with the lean phenotype dominate over bacteria associated with obesity, as co-housing mice having “lean” microbiota with mice having “obese” microbiota prevented obesity development and obesity-associated metabolic syndrome [170]. Moreover, FMT from healthy lean donors to obese patients improved insulin sensitivity [170].

### 3.1. Adipokines

Presently, it has become clear that, in addition to their role in energy storage and adaptive thermogenesis, white (WAT) and brown (BAT) adipose tissues are endocrine organs. WAT and BAT communicate with other organs to regulate metabolism by secreting adipokines and batokines, respectively, signaling types of lipids (lipokines) and exosomal microRNAs (miRNAs) [171,172]. Especially white adipose tissue is a hormonal organ that produces biologically active adipokines, such as adiponectin (APN), IL-1, IL-6, IL-8, IFN-γ, TNF-α, leptin apelin, chemerin, and resistin. Adipokines can regulate metabolic homeostasis and influence immune function [173].

Subjects with SO have elevated plasma levels of pro-inflammatory adipokines [24], which are inversely correlated with muscle strength in these people [24,174]. These substances also suppressed muscle regeneration and promoted atrophy [175,176].

Leptin is produced primarily by adipocytes and is directly related to whole-body obesity [177]. Other tissues, including the stomach, brain, skeletal muscles, and bones, can produce leptin in much lesser amounts [177]. Leptin is a pro-inflammatory adipokine and plays a vital role in modulating the immune response [177].

In rodents, leptin causes an increase in skeletal muscle mass and the size of muscle fibers [178,179]. On the other hand, increased leptin levels possibly produced in intermuscular adipose tissue in older animals have been associated with ectopic myositis and muscle atrophy [180].

In a human study in older subjects, leptin levels were negatively associated with skeletal muscle density [181]. The results of the above studies would suggest a significant role of infiltration of muscles with fat, which could be the source of excess leptin. In a prospective study of older adults, serum leptin concentrations were positively associated with muscle weakness [182]. These data confirm an association between higher leptin levels and decreased muscle quality and function in the older adults, but not necessarily skeletal muscle mass. A recent study found that plasma leptin levels in older women were positively correlated with BMI and negatively with skeletal muscle index (SMI) as an indicator of sarcopenia. The authors concluded that leptin might play a role in the pathogenesis of SO [183].

Adiponectin has initially been identified as a protein secreted by adipose tissue, but it is now known that it can be produced in many tissues, including skeletal muscle. It is an anti-inflammatory adipokine that increases insulin sensitivity in obese animals and humans [184].

It has been shown that adiponectin, possibly of muscle origin, can regulate myogenesis by influencing the proliferation of and differentiation of muscle cells precursors. Therefore, a key role for adiponectin in maintaining the standard structure and function and regeneration of skeletal muscles has been postulated [185,186,187,188,189,190]. Moreover, adiponectin blocked accelerated degradation of skeletal muscle proteins in cultured myotubes by upregulating IRS-1/Akt signaling. Adiponectin was also able to block the expression of TNF-α in adipocytes [191]. The results of the study on the level of adiponectin in individuals with age-related sarcopenia are inconclusive. Although studies by Can et al. [192] showed that patients with senile sarcopenia had significantly lower plasma levels of adiponectin, several other studies showed a relationship between high levels of adiponectin and low muscle density and the incidence of sarcopenia [193,194].

Resistin is a pro-inflammatory adipokine secreted by adipose tissue and immune cells infiltrating adipose tissue [195]. It has been suggested that the resistin is the link between VO and T2D [195]. The resistin/IGF-1 ratio decreases in older people, correlated with lower muscle strength in men [174]. An inverse relationship was also observed between this index and the density of skeletal muscles [196]. Resistin is responsible for suppressing myogenesis, particularly in old skeletal muscle [197,198]. In the culture myotubes from the older adults, incubation with resistin at concentrations corresponding to an older age had a negative effect on myogenesis, indicating greater sensitivity of the muscles of the older adults to resistin [196].

### 3.2. Myosteatosis

Skeletal muscle and bone share a common embryological origin from the mesoderm cell population. Muscle and bone are mechanoresponsive tissues, and the mass and function of both tissues decline with age, both accompanied by accumulation of adipose tissue. Apart from the ageing process, fat accumulation in both tissues is facilitated by obesity, lack of exercise, deficiency of sex hormones, and glucocorticoid exposure [26,27].

Fats can build up in the muscle fibers themselves, called intramuscular fat (IMC), but also between skeletal muscle bundles and below the muscle fascia, called intermuscular fat (IMAT) [26,199]. Fat infiltration (myosteatosis) contributes significantly to the deterioration of muscle function with age [199]. Increased IMAT leads to impaired contractility of skeletal muscles and their metabolic function [200]. Myosteatosis leads to metabolic dysfunction via lipotoxicity and insulin resistance. Furthermore, it has been associated with inflammation and could damage muscle function and quality [26,199,201]. It was recently shown that inhibition of Yap impairs fatty acid oxidation and leads to lipotoxicity in skeletal muscle [167]. Type I fibers (slow-twitch oxidation fibers) collect more lipids with age in humans than type II fibers [202]. The accumulation of adipose tissue in the skeletal muscles can support the conversion of type II fibers to type I and reduce skeletal muscle strength [203].

It should also be noted that intramuscular fat also can secrete pro-inflammatory adipokines, contributing to systemic inflammation and affecting skeletal muscle metabolism [103,204]. It has also been suggested that muscle stem cells may be one of the factors responsible for the accumulation of adipocytes. A type of stem cell other than the satellite cell population has been described. Those cells known as fibro/adipogenic progenitors (FAPs) or mesenchymal interstitial cells are multi-potent progenitors and can differentiate, under certain conditions, such as muscle damage, unlike satellite cells not into myoblasts but adipocytes [205]. FAPs are critical regulators of muscle regeneration, but in pathological situations, such as obesity, they can cause chronic inflammation, fibrosis, and intramuscular fat accumulation in skeletal muscle [205,206]. In obesity, adipokines released mainly from visceral WAT increase FAP adipogenesis, while substances released from myofibers inhibit it [206].

### 3.3. Bone Marrow Adipose Tissue

Bone marrow adipose tissue (MAT) is different from peripheral adipose tissue in terms of location, properties, and function. It is in direct contact with the bone tissue in the bone marrow. Since MAT adipocytes are in a spatially defined range, their expansion can only occur at other cells’ expense.

It is now believed that MAT is not only a regulator of bone metabolism through its paracrine action but may also influence the metabolism of the entire organism [207].

The studies of Krings et al. [208] suggest that the MAT may consist, under normal physiological conditions, predominantly of adipocytes with a metabolic phenotype that combines both BAT and WAT characteristics, which suggests that the adipose tissue in the marrow is similar to the third “beige” type. The so-called “whitening” of these adipocytes, the loss of BAT-like features with ageing, obesity, and other metabolic disturbances, may contribute to adverse changes in the bone marrow environment, supporting bone remodeling [207,208].

Like peripheral adipose tissue, MAT has endocrine functions contributing markedly to adipokines’ local and systemic secretion, particularly adiponectin and leptin [209,210]. As obesity develops, the increased locally released leptin levels from MAT stimulate mesenchymal stem cells (MSCs), acting through the leptin receptor (LepR) to inhibit osteogenesis and promote adipogenesis. As a result of this phenomenon, augmented leptin secretion creates a positive feedback loop that enhances adiposity in the bone marrow [211]. It should also be noted that the increased accumulation of fat in the bones may inhibit of the release of factors acting anabolic on the muscles, such as osteocalcin and IGF-1 [212]. Recently, it has been shown that osteocalcin is essential to prevent age-related muscle loss in mice [213].

## 4. Physical Exercise as a Method of Preventing Sarcopenia

The primary method of preventing and inhibiting the progression of age-related sarcopenia and SO is physical activity [214,215,216,217]. Lack of physical activity in old age is an important risk factor for sarcopenia [214]. Nevertheless, the mechanisms by which exercise can slow down sarcopenia and obesity are complex (for review see, Lazarus and Harridge, 2017; Degens, 2019) [4,218]. Exercise is critical for maintaining a healthy energy balance, and combined with a low-calorie diet, exercise-related energy expenditure can result in a negative energy balance. Exercise is a potent anabolic stimulus and also can improve muscle strength, gait, balance, and aerobic capacity [214,215,216,217].

Resistance training is considered an important strategy to counter sarcopenia; they promote satellite cells activation and proliferation and enhance muscle protein synthesis while inhibiting their breakdown, resulting in increased skeletal muscle mass and strength [219]. Resistance exercise promotes mTOR signaling, which is responsible for the changes in protein synthesis, autophagy, and expression of peroxisome proliferator alpha coactivator 1 (PGC-1) ribosome biogenesis that this exercise elicit. It has been known for some time that the Hippo signaling pathway, and in particular YAP protein, participates in response to and conducting mechanical stimuli. Cellular localization of YAP and its regulatory activity depends on the type, and mechanical stiffness of cell surroundings [220]. Some data point out a decisive influence of the change of cell shape as a triggering stimulus of YAP protein activity what in the case of muscle might be of particular importance, especially having in mind the impact of resistance exercises on muscle physiology [221]. The most convincing evidence that YAP activation might be stimulated by physical stress has presented Aragona et al. [222], showing that cyclic stretching of cells in culture resulted in YAP nuclear translocation. To well-known and scientifically documented cellular signal transduction systems involved in the regulation of muscle hypertrophy (IGF-1-PI3K-Akt-mTOR) and their atrophy (Myostatin-Smad3) one can add a Hippo pathway as an important additional mediator of balance between development and differentiation or atrophy and muscular tissue decay as it has been shown above.

Systematic resistance exercises increase the size of muscle fibers, especially fast-twitch fibers [223]. Resistance exercise is widely recommended to improve muscle mass and skeletal muscle function in the older adults [214,215,216,223]. Increasing the intensity of resistance training and involving larger muscle groups appears to yield more significant effects [216,217,224]. As skeletal muscle hypertrophic potential decreases in old age, it is recommended that patients begin resistance exercises as early as possible [224,225]. Resistance exercises, despite their benefits, have some drawbacks: they can increase the risk of injury, and the range of repetitions can cause boredom and increase the risk of quitting training [217]. Resistance exercise’s impact on body composition and skeletal muscle function in older persons with sarcopenic obesity has received relatively little attention [226]. Despite this, most published evidence indicates that resistance exercise effectively increases body composition, muscle strength, and physical performance in these individuals [227,228,229].

There is less convincing evidence of the effectiveness of other types of exercise [216].

The primary purpose of aerobic exercise in the older adults is to increase/maintain the aerobic capacity of their skeletal muscles [230]. Low—moderate-intensity physical activities (so-called: aerobic exercises) in general have a far lesser effect on the increases of muscle mass than resistance training, but first of all, it is potent to enhance cardio-vascular heath, muscle oxidative capacity, exerts an anti-inflammatory effect, reduces oxidative stress and insulin resistance [231,232] and plays an important role in the control of body mass. They may also inhibit the release of myostatin (MSTN) [200,233]. In addition, aerobic training may have a beneficial effect on maintaining the correct mass of adipose tissue and counteract the development of obesity [234]. Chen et al. [227] demonstrated that aerobic training significantly reduced total fat and visceral adipose tissue in subjects with SO. Interestingly, a more substantial effect was observed after the combination of aerobics and resistance training.

The theoretical foundation for mixing resistance training, walking, aerobic training, balance training, and other types of training in multimodal exercise therapy is well-founded. [235]. Existing research, albeit still in its infancy, appears to back up these assertions [216,235].

A new blood flow restriction (BFR) approach, which partially restricts arterial inflow while totally limiting venous outflow in the muscles during exercise, offers an intriguing alternative [236,237]. During low-intensity training, this approach allows for a significant gain in skeletal muscle strength. [236]. However, some experts are concerned about the method’s potential adverse side effects and propose that training only occurs under the supervision of qualified staff [236,237].

Alternative approaches are offered since some older adult persons are unable to exercise for various reasons. Preliminary findings suggest that whole-body vibration (WBV) and whole-body electromyostimulation (WB-EMS) may be effective in the treatment of sarcopenia, but more research is needed. [238,239].

Although physical activity plays an important role in slowing down the process of sarcopenia in ageing people, even a high dose of physical activity cannot stop the ageing-related loss of muscle mass, their force and power generating capabilities in humans (see, e.g., Lazarus and Harridge, 2017) [218].

## 5. A Role for Myokines in Sarcopenia: Cross-Talk between Muscle and Adipose Tissue

Research has consistently shown that regular exercise provides remarkable health benefits, plays a role in preventing or reducing the effects of chronic disease, slowing biological ageing, and prolonging life [240]. The mechanisms of these health benefits are complex but can be at least partly attributed to bioactive substances released into the circulation during exercise [241,242]. It is now widely accepted that skeletal muscles, besides their primary functions, play the role of endocrine organs, producing and releasing cytokines and other peptides, exerting autocrine, paracrine and hormonal effects on various tissues [241].

**Figure 6:**
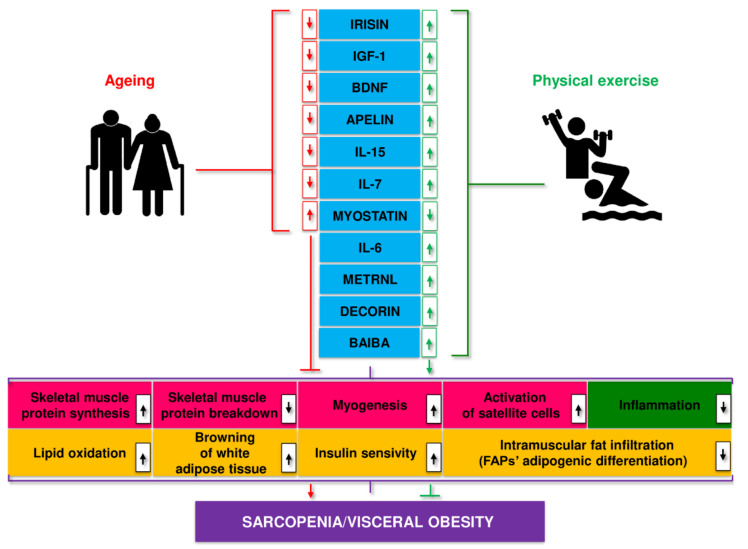
Myokines linked to age-related changes, their release during exercise, and putative mechanisms of action. More information on the listed myokines is described in specific paragraphs.

Disorders in myokine secretion may play a role in the pathogenesis of age-related and metabolic diseases, including obesity, T2D, sarcopenia, and SO [111,243,244]. Ageing leads to a decrease in the secretion of most myokines, including apelin, BAIBA, decorin, IGF-1, IL-15, irisin, sesterin, SPARC, while the secretion of myostatin increases. These processes were partially reversed by regular physical activity (Figure 6) [245].

Muscle hypertrophy is a critical adaptation to regular exercise, particularly resistance training. This effect is likely mediated by insulin-like growth factor 1 (IGF-1) generated in muscles during exercise [49]. Other myokines produced by exercise also appear to positively affect the proliferation of satellite cells and muscle hypertrophy [246,247]. Reduced myostatin levels, a muscle growth inhibitor, and altered control of myostatin activity in exercising skeletal muscles, presumably influenced by other myokines, may also contribute to muscle hypertrophy. Research in recent years suggests that myokines may act as diagnostic biomarkers and therapeutic targets in sarcopenia and SO [147,244,245,248,249,250].

### 5.1. IGF-1

IGF-1 is a key growth factor that controls the anabolic and catabolic pathways in skeletal muscle and thus plays a crucial role in muscle growth, differentiation and regeneration [49]. In adults, IGF-1 is primarily synthesized in the liver and acts as a systemic growth factor but is also released from skeletal muscle and acts in auto and paracrine ways. IGF-1 has an anabolic effect in skeletal muscle through the PI3K/Akt/mTOR and PI3K/Akt/GSK3β pathways. Via PI3K/Akt, it can also inhibit FoxOs and thus the transcription of E3 ubiquitin ligases regulating protein breakdown via the ubiquitin-proteasome system (UPS) [49]. Autophagy is possibly also inhibited by IGF-1 via mTOR and FoxO signals. IGF-1 also stimulates the activation of satellite cells, contributing to muscle hypertrophy and inhibiting atrophy [49]. In skeletal muscles, there are several IGF-1 isoforms with various degrees of potency in promoting hypertrophy. IGF-1 levels and IGF-1R signaling are suppressed in many chronic disorders, including age-related sarcopenia, possibly causing muscle atrophy due to the combined effects of altered protein synthesis, UPS activity, autophagy, and impaired muscle regeneration [49,251,252].

Plasma IGF-1 decreases with age, and low IGF-1 levels are associated with sarcopenia risk [253,254,255,256,257,258,259]. Some authors suggested that low IGF-1 could be a promising biomarker for sarcopenia [260,261,262]. The research of Poggiogalle et al. [263] showed that the impairment of the GH/IGF-1 axis might be linked to an increased risk of developing sarcopenic obesity. Interestingly, GF mice have decreased IGF1 and PGC1α expression [124].

Various forms of physical activity led to a significant increase in IGF-1 levels, even in older adults of both sexes and resistance training was particularly effective [258,261,264,265,266,267,268,269]. These observations suggest that a decreased level of IGF-1 is associated with the risk of developing sarcopenia, especially sarcopenic obesity.

### 5.2. Myostatin

MSTN was the first myokine discovered, although it was not initially known by that name [270]. MSTN is the myokine with the most well-documented effects on muscle and adipose tissue. MSTN, which is also known as growth differentiation factor 8 (GDF-8), belongs to the transforming growth factor β (TGF-β) superfamily, is expressed primarily in skeletal muscle, and to a lesser extent in adipose tissue and cardiac muscle, and acts as a negative regulator of the muscle mass growth and development [36,270,271,272].

MSTN inhibits skeletal muscle protein synthesis by binding to the activin type IIB receptor (ActRIIB) and the subsequent phosphorylation of Smad2 and Smad3 [273]. This process leads to the activation of genes involved in the degradation of muscle proteins and the simultaneous inhibition of protein synthesis by inhibiting the IGF-1/Akt/mammalian target pathway of rapamycin (mTOR) [273,274]. MSTN also facilitates muscle atrophy via the forkhead box protein O1 (FoxO1) pathway and, by inhibiting GLUT4 and AMP-activated protein kinase (AMPK), decreases glucose uptake in skeletal muscle [275,276,277,278]. These effects are inhibited by endogenous follistatin, which activates the Akt-mTOR pathway, stimulates protein synthesis and functions as a pro-hypertrophic signal. Different types of exercise were shown to increase follistatin plasma levels [279].

MSTN is upregulated in obesity animal models of obesity, and elevated myostatin levels have been observed in obesity in humans. Regular physical activity inhibits MSTN expression in skeletal muscles in obese persons, but a lower amount of mRNA has also been detected in healthy people after one intensive exercise [270,280,281,282,283,284]. It has also been shown that MSTN positively regulates adipogenesis [285]. MSTN levels are positively associated with IMAT, indicating a potential role for this myokine in the development of myosteatosis [200]. Elevated MSTN levels are also associated with increased insulin resistance [286,287,288]. Amor et al. [284] observed a positive correlation of circulating myostatin concentration with indices of insulin resistance and a negative correlation with indices of insulin sensitivity.

Aged muscle has enhanced MSTN signaling, and the observed increase in MSTN levels with ageing may be partially responsible for the age-related reduction in skeletal muscle mass and strength [262,289,290,291]. It has also been indicated that MSTN may induce irisin biosynthesis inhibition and contribute to a rise in fat mass and a decline in muscle mass, which is especially harmful to the older adults, predisposing them to SO [271].

### 5.3. Irisin

Irisin first identified as peroxisome proliferator-activated receptor γ coactivator-1α (PGC-1α)-dependent myokine is released into the circulation by cleavage of the type III fibronectin domain (FNDC5) protein bound to the skeletal muscle membrane in response to exercise or muscle shivering, causing browning and regulating thermogenesis in white adipose tissue [292,293].

In humans, resting levels of irisin decline with age [294,295]. In older rats, irisin expression was lower than in young rats [296]. Research in recent years suggests that irisin may be used as a biomarker of sarcopenia and SO and be used in early screening for age-related muscle changes [295,297,298,299,300].

Most studies have shown that various forms of exercise increase irisin plasma levels and its expression in muscle in humans and animals [294,301,302,303,304,305,306,307,308,309,310,311,312,313,314,315,316,317,318,319], but some studies have contradicted this observation [320,321,322]. A cross-sectional study found that physically active people had higher irisin levels than inactive people [323].

Irisin induces the expression of promiogenic genes in myotubes, increases myogenic differentiation and myoblast fusion [324,325]. Irisin injection in mice improves regeneration, induces hypertrophy and reduces protein degradation due to activation of satellite cells and increased protein synthesis [324]. Irisin had an anti-atrophic effect on C2C12 myotubes treated with dexamethasone (DEX), a recognized inducer of muscle atrophy, inhibiting FoxO-dependent ubiquitin-proteasome overactivity [326].

Inhibition of MSTN has been demonstrated to raise irisin levels in animal studies [327]. Irisin has also been linked to decreased adipose tissue mass and increased insulin sensitivity [313,328].

### 5.4. Meteorin-like Factor (metrnl)

Meteorin-like factor (metrnl) is a new myokine capable of browning white adipose tissue and reducing insulin resistance. Several recent studies have confirmed the involvement of metrnl as an immunological/metabolic regulator of adipose tissue. Cold temperatures and exercise enhance metrnl expression in skeletal muscle and adipose tissue and increase plasma levels. Elevated metrnl levels promote the browning of white adipocytes. Interestingly, metrnl does not appear to operate directly on adipocytes but rather through cells of the immune system that infiltrate adipose tissue [329,330].

According to the findings of Baht et al. [329], metrnl is required for proper muscle regeneration. Metrnl-deficient mice had defective muscle regeneration related to a decreased infiltration of various cells of the immune system and an inability to shift to an anti-inflammatory phenotype. Metrnl was shown to act directly on macrophages via a Stat3-dependent mechanism. This causes an anti-inflammatory response and the activation of IGF-1, which stimulates muscle satellite cells and myogenesis [329,330].

### 5.5. Brain-Derived Neurotrophic Factor (BDNF)

BDNF is a neurotrophin found mainly in the brain and skeletal muscles, which plays a role in learning and memory [331]. Patients with neurodegenerative diseases have low plasma levels of BDNF [332]. Low levels of BDNF are also found in patients with obesity and T2D [333,334,335]. Exercise increases the expression of BDNF in human skeletal muscles [336], and resistance exercise increases BDNF plasma levels. BDNF affects myogenesis in skeletal muscle and activation of satellite cells [337,338]. BDNF is expressed in satellite cells in adult skeletal muscle. Muscle damage causes an increase in BDNF expression, which coincides with satellite cell activation and proliferation, implying that BDNF may play an essential role in modulating satellite cell responses to injury and in the regeneration process [339].

Overexpression of BDNF promotes genes associated with fast muscle type and increases the number of glycolytic fibers [340].

Physical activity increases plasma/serum BDNF level both in young healthy people [341,342] as well as in patients with various neurological disorders, including Parkinson’s diseases patients [343,344]. Plasma levels of BDNF were found significantly higher in non-frail than pre-frail older women, and physical therapy intervention increased plasma BDNF levels in both groups [345]. Low BDNF levels have been associated with decreased physical function and the prevalence of severe sarcopenia and frailty in Japanese patients undergoing maintenance hemodialysis [346]. The systemic review has shown that exercise increases peripheral BDNF levels in healthy older adults and older adults with different pathologies [347]. It has been suggested that BDNF signaling may play an essential role in regulating neuromuscular function during ageing, which may have implications for the pathogenesis of sarcopenia and SO [244].

### 5.6. Fibroblast Growth Factor 21 (FGF21)

Fibroblast growth factor 21 (FGF21) is the only one from the FGF superfamily that does not participate in the processes of proliferation and differentiation of cells and tissues but plays a vital role in regulating metabolic activities [348,349]. While the liver is the primary source of FGF21, it is now known that adipocytes, and especially myocytes, are an essential source of FGF21. FGF21, released by myocytes upon exposure to cold or exercise, protects the body against obesity and insulin resistance, in part by antagonizing adipokines released by adipose tissue [348,349]. FGF21 is a crucial regulator of the differentiation of WAT to beige adipocytes through UCP1-dependent and -independent mechanisms [350]. In addition, upon exposure to cold or exercise, there is increased thermogenesis in skeletal muscle and adipose tissue through FGF21-induced upregulation of the local coactivator of the peroxisome proliferator-activated (PGC) -1-alpha gamma receptor [350]. Transferring the FGF21 gene to healthy mice using adeno-associated viral vectors prevented age-related weight gain and insulin resistance and promoted healthy ageing [351]. Kim et al. [352] demonstrated that FGF21 deficiency exacerbated obesity-induced inflammation and atrophic responses in skeletal muscle of obese mice, and FGF21 treatment protected against inflammation-induced atrophy via the AMPK pathway. Liu et al. [353] have shown that FGF21 induces myoblast differentiation and can act as a switch for molecular transformation from anaerobic to aerobic fibers via the FGF21-SIRT1-AMPK-PGC1α axis. FGF21 also inhibits the accumulation of fat in the muscles [354]. Therefore, studies indirectly show that sarcopenia and SO may be correlated with the FGF21 disorder.

### 5.7. β-Aminoisobutyric Acid (BAIBA)

BAIBA, a catabolite of thymine and valine metabolism, is a non-proteinogenic amino acid [355]. Recently BAIBA has been identified as a potential myokine, secreted during skeletal muscle contraction through the action of the 1α coactivator of the peroxisome proliferator-activated receptor (PGC-1α) [356]. BAIBA plasma levels increase in response to regular exercise in humans and animals [355,356]. BAIBA is involved in the glucose homeostasis beiging of subcutaneous white adipose tissue, suppressing inflammation in skeletal muscle and adipose tissue, and decreasing adipose tissue mass [356,357,358,359,360,361]. Plasma BAIBA levels are higher in young people than in older people [362,363]. It was revealed that the protective effect of BAIBA was lost with age, not due to loss of the muscle capacity to produce BAIBA but likely to reduced Mas-Related G Protein-Coupled Receptor Type D expression with ageing [364].

### 5.8. Apelin

Apelin is the endogenous ligand of the specific APJ receptor, the G protein-coupled receptor. They form the apelin/APJ system, which is widely distributed in various tissues [365]. Apelin is an adipokine and myokine; its release from muscles is regulated by exercise. Training increases muscle and plasma apelin levels and improves metabolism in animals and people with obesity or T2D [366,367]. There is a significant age-related decline in apelin levels in rodents and humans, partially prevented by exercise [260,368,369]. In mice deficient in apelin or its receptor, especially pronounced age-related changes in muscle function were demonstrated [260,368,369]. Apelin restoration counteracted these changes, improved muscle function by acting anti-inflammatory, and increased the regenerative abilities of muscles by affecting satellite cells [260,368,369]. These results indicate that apelin can be a biomarker of early sarcopenia and a pharmacological agent against sarcopenia.

### 5.9. Decorin

Decorin is a myokine secreted in skeletal muscle during muscle contraction, and its level increases in response to exercise, particularly resistance exercise [370,371,372]. The expression of decorin mRNA in human skin fibroblasts significantly decreases with age [373]. Removal of decorin in mice can impair glucose tolerance with increased VAT weight and leptin levels [374]. Decorin is involved in stimulating muscle growth in part by inhibiting myostatin [370,375]. Increased expression of decorin in skeletal muscle in mice promoted the expression of Myod1, follistatin and the promiogenic factor Mighty, downregulated by myostatin. At the same time, overexpression of decorin in skeletal muscle reduced the muscle-specific ubiquitin ligases atrogin1 and MuRF1 involved in atrophic pathways. In patients with liver cirrhosis, the level of serum decorin was significantly related to skeletal muscle mass and was an independent factor in the absence of skeletal muscle atrophy [376]. Decorin may be a potential therapeutic target in sarcopenia, especially in SO.

### 5.10. Il-6

Interleukin-6 (IL-6), the prototypical myokine, is released in large amounts into the circulating blood during skeletal muscle contraction, depending on the exercise intensity [377]. IL-6 released during exercise may act locally, regulate muscle metabolism, exert endocrine effects on distant tissues and influence glucose homeostasis and body lipid metabolism [378,379].

IL-6 may have both pro-inflammatory and anti-inflammatory effects and may also have an anabolic or catabolic effect on muscles. The final effect may depend on the cytokine release mode, target structure, and simultaneous presence of other cytokines.

Several studies have suggested that IL-6, particularly in chronic diseases, may exacerbate muscle atrophy [380,381,382]. Elevated levels of IL-6 also play a role in age-related low-grade inflammation and contribute to the development of sarcopenia [96,97,383]. IL-6 can promote skeletal muscle atrophy by diminishing muscle anabolism and directly mediating muscle catabolism [384]. Madaro et al. [385] observed that denervation-activated FAPs exhibited sustained STAT3 activation and increased IL-6 secretion in a mouse model, which promoted skeletal muscle atrophy. As age-related denervation would play a role in the pathogenesis of sarcopenia, this mechanism could contribute to this process [37]. However, some observations indicate that the action of elevated IL-6 alone is not sufficient to induce skeletal muscle atrophy. For example, in the experimentally induced sepsis model, IL-6 knockout mice did not have increased skeletal muscle catabolism compared to wild-type mice [386]. It has been proposed that long-term exposure to elevated levels of IL-6 and synergistic interaction with other pro-inflammatory cytokines are necessary to induce such catabolic effects [109].

IL-6 is mainly known as a pro-inflammatory cytokine, but when released during muscle contraction, it is involved in the anti-inflammatory effects of exercise, inhibiting the production of pro-inflammatory cytokines [378,379].

IL-6 stimulates glycogenolysis and lipolysis in skeletal muscle through AMPK activation. It was observed that exercise-induced rise in IL-6 levels enhances GLUT4 expression and insulin sensitivity in skeletal muscle [387]. IL-6 has also been involved in browning WAT adipocytes [388,389].

It has been demonstrated in animal and human studies that IL-6 plays an essential role in muscle hypertrophy in response to exercise and muscle regeneration after muscle damage [247,377,390,391,392]. IL-6 seems to be a vital regulator of muscle satellite cell proliferation and myogenic differentiation [247,391,393,394] and can also activate the myotube mTOR pathway and myotube protein synthesis [395]. Taniguchi et al. [396] found that gp130, an IL-6 cytokine coreceptor, stimulates YAP and Notch, transcription regulators that control tissue growth and regeneration, independently of the gp130 STAT3 effector, which could explain the effects of IL-6 released during exercise.

The production of IL-6 in response to exercise in skeletal muscle seems to depend on the Ca^2+/^calcineurin signaling pathway for IL-6 induction [397,398]. Increased Ca2+ concentration leads to increased IL-6 mRNA concomitantly with a down-regulation of TNF-α [397].

During exercise, only IL-6 is secreted, and TNF-α is essentially unchanged. The only extreme marathon-like physical exercise produced a slight increase in TNF-α level [399]. It has been suggested [109,205,377] that the positive effect of IL-6 is related to its transient production and short-term action in the absence of pro-inflammatory cytokines. On the other hand, the long-lasting effects of elevated levels of IL-6 in chronic inflammation and the simultaneous action of pro-inflammatory cytokines such as TNF-α make IL-6 pro-inflammatory, promote muscle catabolism and disturb immune homeostasis [377]. Such a situation may occur in the context of sarcopenia, with impaired muscle function and low physical activity [109].

### 5.11. IL-7

Interleukin-7 (IL-7) is produced mainly by stromal cells in lymphoid organs such as bone marrow, thymus, spleen and lymph nodes, but it can also be produced by non-lymphoid tissues including intestine, skin, lungs and liver [400]. Due to its expression and secretion from skeletal muscle cells, IL-7 is now considered a myokine [401]. Skeletal muscle IL-7 expression is increased with regular training [401,402]. IL-7 plays an essential role in myogenesis and may influence the differentiation of satellite cells into fully developed skeletal muscle cells [111,401,403]. Ageing, HFD, and obesity all reduce IL-7 in muscle and serum [404,405]. Interestingly, exercise can counteract this, which would indicate skeletal muscles as an important source of IL-7 [404,406].

### 5.12. IL-15

Recently, the role of a myokine regulating metabolism has been proposed for Interleukin 15 (IL-15). IL-15 is a member of the IL-2 superfamily and is implicated in skeletal muscle-adipose tissue cross-talk [407,408,409,410]. IL-15 is released by physical exercise and can reduce visceral adipose tissue mass in humans and rodents [407,408,409,410,411,412]. The increase in plasma IL-15 levels after exercise appears to be pulsatile and short-lived [412]. In contrast, HFD-fed animals were found to have decreased levels of IL-15 in plasma and muscles [413].

IL-15 may counteract HFD-induced obesity, insulin resistance, and fatty liver [408,414,415,416,417,418,419]. IL-15 inhibits the accumulation of lipids in preadipocytes and stimulates the secretion of adiponectin, which indirectly reduces adipose tissue mass [407,408,409]. Exercise appears to control IL-15 expression through skeletal muscle AMP-activated protein kinase (AMPK) [411,420], and transgenic mice with functionally inactive AMPK had decreased levels of both plasma IL-15 concentration and IL-15 mRNA [411]. IL-15 is an anabolic factor involved in regulating skeletal muscle growth [421,422]. IL-15 inhibits protein degradation in muscle and prevents intramuscular fat infiltration, possibly affecting FAPs [423,424,425]. This myokine stimulates the proliferation of FAPs and, at the same time, inhibits their adipogenic differentiation, probably through the induction of desert Hedgehog (DHH) signaling [425,426].

Some studies have suggested that low IL-15 levels may play a role in sarcopenia’s pathogenesis [427,428]. Studies in rodents have shown that the levels of IL-15 in muscle and serum decline gradually with age [429,430]. In humans, an age-dependent decrease in IL-15 levels also has been observed [406,431] AMPK activity declines with ageing, which is another mechanism potentially responsible for disrupted IL-15 signaling in ageing muscle [411,432].

The possible modification of IL-15 levels through regular exercise and preventing them from decreasing with age may be significant in terms of the effect of this cytokine on the maintenance of immune function and the simultaneous stimulation of myogenesis, and prevention of abnormal distribution of adipose tissue.

### 5.13. Other Myokines

In addition to these myokines, there are many others, such as leukemia inhibitory factor (LIF), musclin, myonectin, matrix metalloproteinase 2 (MMP-2), monocyte chemoattractant protein 1 (MCP-1), follistatin-like protein 1 (FSTL-1), and mitsugumin 53 (MG53), secreted protein acidic and rich in cysteine (SPARC), angiopoietin-like 4 (ANGPTL4), sestrin, Bone Morphogenetic Protein 7 (BMP-7) [111,433,434,435]. They are released in response to exercise; many counteract visceral obesity and IR and play a role in myogenesis [111,433,434]. Their role in sarcopenia and SO requires further research.

## 6. Conclusions

Obesity and ageing are major health costs for the world’s adult population. Both factors increase the risk of developing related metabolic disorders. Sarcopenic obesity is defined by the presence of both sarcopenia and obesity. It has a significant impact on the health of the older adults. Visceral adipose tissue (VAT) becomes dysfunctional during obesity and ageing and plays an essential role in its pathophysiology. Changes in VAT related to obesity and ageing are due in part to chronic local inflammation. The gut–muscular axis may be involved in the pathogenesis of sarcopenia and SO. Recent research supports the notion that dysbiosis may play a role in the onset and progression of sarcopenia and SO. Due to its complex pathophysiology, there is disagreement and difficulty in defining, diagnosing, and treating SO. Numerous studies have shown that myokines, released by skeletal muscles, play a vital role in controlling muscle hypertrophy, function, and metabolic balance. Myokine dysfunction can trigger and exacerbate the pathogenesis of underlying metabolic and age-related disorders, such as obesity, sarcopenia, T2D, and SO. Physical activity, in addition to proper nutritional supplementation, is the only practical approach to delay the onset of, and to treat, sarcopenia, especially SO. Although physical activity cannot fully inhibit the process of sarcopenia and the age-related deterioration of muscle function, it can clearly delay the onset of sarcopenia and attenuate its rate. This is why physical training involving both resistance and endurance training in an appropriate dose are highly recommended to practice even at an advanced age.
